# 5-(4,5-Diiodo-1,3-dithiol-2-yl­idene)-4′,5′-bis(methyl­sulfan­yl)-2,2′-bi-1,3-dithiol-4(5*H*)-one

**DOI:** 10.1107/S160053680904032X

**Published:** 2009-10-10

**Authors:** Kazumasa Ueda, Kenji Yoza

**Affiliations:** a Division of Applied Science and Fundamental Engineering, Faculty of Engineering, Shizuoka University, Johoku 3-5-1, Hamamatsu, Shizuoka 432-8561, Japan; b Bruker AXS Co. Ltd., Moriya-cho 3-9, Kanagawa-ku, Kanagawa, Kanagawa 221-0022, Japan

## Abstract

The mol­ecular framework of the title compound, C_11_H_6_I_2_OS_8_, is almost planar [maximum deviation = 0.057 (5) Å] except for the two methyl­sulfanyl groups, which are twisted relative to the mol­ecular skeleton, with C—C—S—C torsion angles of 49.74 (22) and 82.91 (21)°. In the crystal, mol­ecules are stacked alternately in opposite orientations, forming a one-dimensional column along the *b* axis. The inter­action between adjacent columns is accomplished through S⋯S [3.4289 (5) Å], S⋯I [3.4498 (4) Å] and O⋯I [2.812 (2) Å] contacts.

## Related literature

For background to tetra­thia­fulvalenoquinone-1,3-dithiol­emethide derivatives, see: Matsumoto *et al.* (2002*a* ▶,*b* ▶; 2003 ▶); Hiraoka *et al.* (2007 ▶); Sugimoto (2008 ▶). For the synthesis, see: Iwamatsu *et al.* (1999 ▶). For background to inter­molecular I⋯O contacts, see: Etter (1976*a* ▶,*b* ▶); Groth & Hassel (1965 ▶); Leser & Rabinovich (1978 ▶). For van der Waals radii, see: Bondi (1964 ▶).
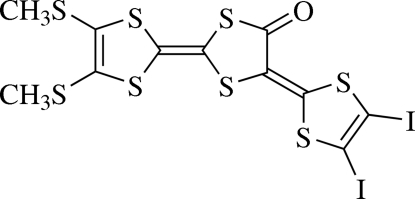


## Experimental

### 

#### Crystal data

C_11_H_6_I_2_OS_8_
*M* *_r_* = 664.44
Monoclinic, 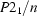

*a* = 7.7642 (14) Å
*b* = 17.652 (3) Å
*c* = 14.124 (3) Å
β = 98.188 (2)°
*V* = 1916.0 (6) Å^3^
*Z* = 4
Mo *K*α radiation
μ = 4.15 mm^−1^
*T* = 93 K
0.10 × 0.07 × 0.03 mm

#### Data collection

Bruker APEXII CCD area-detector diffractometer
Absorption correction: multi-scan (*SADABS*; Sheldrick, 1996 ▶) *T*_min_ = 0.682, *T*_max_ = 0.886
11092 measured reflections
4403 independent reflections
3764 reflections with *I* > 2σ(*I*)
*R*_int_ = 0.030

#### Refinement

*R*[*F* ^2^ > 2σ(*F* ^2^)] = 0.025
*wR*(*F* ^2^) = 0.051
*S* = 0.99
4403 reflections
201 parameters
H-atom parameters constrained
Δρ_max_ = 0.72 e Å^−3^
Δρ_min_ = −0.56 e Å^−3^

### 

Data collection: *APEX2* (Bruker, 2006 ▶); cell refinement: *SAINT* (Bruker, 2006 ▶); data reduction: *SAINT*; program(s) used to solve structure: *SHELXS97* (Sheldrick, 2008 ▶); program(s) used to refine structure: *SHELXL97* (Sheldrick, 2008 ▶); molecular graphics: *XSHEL* (Bruker, 2002 ▶); software used to prepare material for publication: *XCIF* (Bruker, 2001 ▶).

## Supplementary Material

Crystal structure: contains datablocks I, global. DOI: 10.1107/S160053680904032X/tk2544sup1.cif

Structure factors: contains datablocks I. DOI: 10.1107/S160053680904032X/tk2544Isup2.hkl

Additional supplementary materials:  crystallographic information; 3D view; checkCIF report

## Figures and Tables

**Fig. 1.**
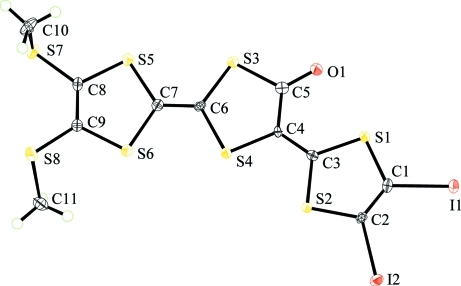
The molecular structure of (I) showing atom labelling and 50% probability displacement ellipsoids for non-H atoms.

**Fig. 2.**
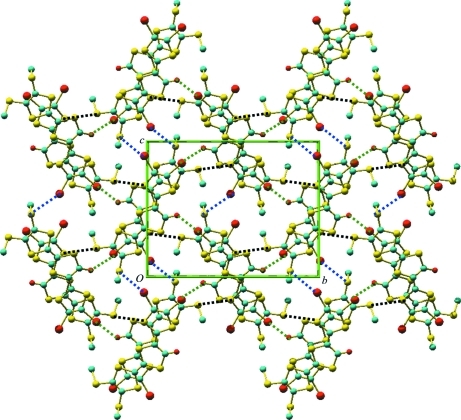
Projection of the crystal packing in (I) down the bc plane. The S···S (black), S···I (blue) and O···I (green) contacts are shown with dashed lines.

## supplementary crystallographic information

### Comment

New donor molecules featuring a skeleton of tetrathiafulvalenoquinone-1,3-dithiolemethide are used for the preparation of charge transfer (CT) salts with magnetic metal anions (Matsumoto *et al.*, 2002a,b, 2003; Hiraoka *et al.*, 2007; Sugimoto 2008). In CT salts these molecules can form unique crystal structures with channels in addition to the usual layer stacking structures as a result of their molecular skeletons and intermolecular S···S contacts. The introduction of iodide atoms as substituents in the molecular skeleton is expected to enhance intermolecular interaction through the formation of S···I and O···I heteroatom contacts. These contacts are of special interest in these structures as they may increase the dimensionality of aggregation in the solid-state. In this connection, the crystal structure of the title compound, (I), was investigated.

The molecular framework of (I), Fig. 1, except for two methylsulfanyl groups, is almost planar. The displacements of atoms S7, S8, I1, and I2 relative to the plane of the skeleton are 0.2056 (17), 0.230 (2), -0.1867 (15) and -0.1274 (18) Å, respectively. The torsion angles of the two methylsulfanyl groups are -49.74 (22)° for C11—S8—C9—S6 and -89.91 (21)° for C10—S7—C8—S5.

In the crystal structure, the molecules are alternatively stacked in opposite orientations to form a one-dimensional column along the *a* axis (Fig. 2). Stacked molecules are separated by interplanar distances greater than 3.54 Å and have fairly poor overlap. However, some effective side-by-side contacts are observed between molecules of adjacent columns. The interaction between adjacent columns is accomplished through contacts between different sulfur atoms [S2···S8^i^ = 3.4289 (5) Å] along the *b* axis, between sulfur and iodide atoms [S7···I2^ii^ = 3.4498 (4) Å] along the *c* axis, and between oxygen and iodide atoms [O1···I1^iii^ = 2.812 (2) Å] along the *b* axis; symmetry operation i: -1/2+*x*, 1/2-*y*, -1/2+*z*; ii: 1+*x*, *y*, 1+*z*; and iii: 1/2+*x*, -1/2-*y*, 1/2+*z*. These distances are shorter than the sum of corresponding van der Waals radii, i.e. 3.60 Å for S···S, 3.78 Å for S···I and 3.32 Å for O···I (Bondi, 1964). An interesting feature of this structure is the fairly shorter intermolecular O···I contacts. Such strong oxygen-halogen interactions have been observed previously (Groth & Hassel, 1965; Etter, 1976a,b). The intermolecular angles are 124.20 (19)° for C5=O1···I1 and 176.17 (10)° for O1···I1—C2 are fairly close to the ideal geometry (120° for C=O···I and 180° for O···I—C) which has been proposed for these types of associations (Leser & Rabinovich, 1978).

### Experimental

Compound (I) was synthesized by a modification of the method used for the preparation of bis(methylsulfanyl)tetrathiafulvalenoquinone-1,3-dithiolemethide (Iwamatsu *et al.*, 1999). Bis(tetraethylammonium)bis(2,3-bis(methylsulfanyl)tetrathiafulvalenyl-6,7- dithiolato)zinc (269 mg, 0.258 mmol) was reacted with 4,5-diiodo-2-methylsulfanyl-1,3-dithiole-2,3-dithiolium tetrafluoroborate (535 mg, 1.10 mmol) in THF-DMF (5:1 = *v*/*v*,) at room temperature under nitrogen and stirring for 12 h. After separation of the reaction mixture by column chromatography on silica gel (eluent: CS_2_) followed by recrystallization from CS_2_/*n*-hexane, bis(dimethylsulfanyl)tetrathiafulvalenothioquinone- 4,5-diiodo-1,3-dithiolemethide (II) was obtained as a dark-green needles in 72% yield. When compound (II) (87 mg, 0.127 mmol) was reacted with mercury(II) acetate (90 mg, 0.282 mmol) in THF-AcOH (5:1 =*v*/*v*), compound (I) was obtained as a dark-red plates in 47% yield by recrystallization from CS_2_/*n*-hexane.

### Refinement

The H atoms were geometrically placed with C-H = 0.98Å, and refined as riding with U_iso_(H) = 1.5U_eq_(C).

### Crystal data

**Table:**

C_11_H_6_I_2_OS_8_	*F*(000) = 1256
*M**_r_* = 664.44	*D*_x_ = 2.303 Mg m^−^^3^
Monoclinic, *P*2_1_/*n*	Mo *K*α radiation, λ = 0.71073 Å
*a* = 7.7642 (14) Å	Cell parameters from 3988 reflections
*b* = 17.652 (3) Å	θ = 2.3–27.5°
*c* = 14.124 (3) Å	µ = 4.15 mm^−^^1^
β = 98.188 (2)°	*T* = 93 K
*V* = 1916.0 (6) Å^3^	Plate, dark-red
*Z* = 4	0.10 × 0.07 × 0.03 mm

### Data collection

**Table:**

Bruker APEXII CCD area-detector diffractometer	4403 independent reflections
Radiation source: Bruker TXS fine-focus rotating anode	3764 reflections with *I* > 2σ(*I*)
Bruker Helios multilayer confocal mirror	*R*_int_ = 0.030
Detector resolution: 8.333 pixels mm^-1^	θ_max_ = 27.6°, θ_min_ = 1.9°
φ and ω scans	*h* = −6→10
Absorption correction: multi-scan (*SADABS*; Sheldrick, 1996)	*k* = −22→22
*T*_min_ = 0.682, *T*_max_ = 0.886	*l* = −18→15
11092 measured reflections	

### Refinement

**Table:**

Refinement on *F*^2^	Primary atom site location: structure-invariant direct methods
Least-squares matrix: full	Secondary atom site location: difference Fourier map
*R*[*F*^2^ > 2σ(*F*^2^)] = 0.025	Hydrogen site location: inferred from neighbouring sites
*wR*(*F*^2^) = 0.051	H-atom parameters constrained
*S* = 0.99	*w* = 1/[σ^2^(*F*_o_^2^) + (0.019*P*)^2^] where *P* = (*F*_o_^2^ + 2*F*_c_^2^)/3
4403 reflections	(Δ/σ)_max_ = 0.006
201 parameters	Δρ_max_ = 0.72 e Å^−^^3^
0 restraints	Δρ_min_ = −0.56 e Å^−^^3^

### Special details

**Table:**

Geometry. All e.s.d.'s (except the e.s.d. in the dihedral angle between two l.s. planes) are estimated using the full covariance matrix. The cell e.s.d.'s are taken into account individually in the estimation of e.s.d.'s in distances, angles and torsion angles; correlations between e.s.d.'s in cell parameters are only used when they are defined by crystal symmetry. An approximate (isotropic) treatment of cell e.s.d.'s is used for estimating e.s.d.'s involving l.s. planes.
Refinement. Refinement of *F*^2^ against ALL reflections. The weighted *R*-factor *wR* and goodness of fit *S* are based on *F*^2^, conventional *R*-factors *R* are based on *F*, with *F* set to zero for negative *F*^2^. The threshold expression of *F*^2^ > σ(*F*^2^) is used only for calculating *R*-factors(gt) *etc*. and is not relevant to the choice of reflections for refinement. *R*-factors based on *F*^2^ are statistically about twice as large as those based on *F*, and *R*- factors based on ALL data will be even larger.

### Fractional atomic coordinates and isotropic or equivalent isotropic displacement parameters (Å^2^)

**Table:**

	*x*	*y*	*z*	*U*_iso_*/*U*_eq_	
I2	−0.12578 (3)	0.013561 (12)	0.111820 (14)	0.01793 (6)	
I1	−0.15011 (3)	−0.203405 (12)	0.164968 (14)	0.01569 (6)	
C9	0.6068 (4)	0.19937 (18)	0.7771 (2)	0.0152 (7)	
C1	−0.0462 (4)	−0.11760 (18)	0.2570 (2)	0.0149 (7)	
C6	0.4087 (4)	0.01803 (18)	0.6428 (2)	0.0136 (6)	
C4	0.2274 (4)	−0.04697 (17)	0.4954 (2)	0.0120 (6)	
C8	0.6498 (4)	0.14725 (18)	0.8460 (2)	0.0154 (7)	
C2	−0.0379 (4)	−0.04383 (18)	0.2381 (2)	0.0143 (7)	
C3	0.1281 (4)	−0.06019 (17)	0.4091 (2)	0.0123 (6)	
C7	0.4882 (4)	0.07062 (18)	0.7018 (2)	0.0142 (7)	
C5	0.2707 (4)	−0.10758 (18)	0.5631 (2)	0.0142 (6)	
S6	0.49464 (10)	0.16647 (5)	0.66722 (6)	0.01662 (17)	
S4	0.30314 (10)	0.04434 (4)	0.52857 (5)	0.01454 (16)	
S2	0.06947 (10)	0.01342 (4)	0.32957 (5)	0.01350 (16)	
S7	0.75758 (11)	0.16499 (5)	0.96192 (6)	0.02020 (18)	
S5	0.59033 (10)	0.05246 (5)	0.81921 (6)	0.01688 (17)	
S3	0.39758 (10)	−0.07854 (5)	0.67194 (6)	0.01666 (17)	
S1	0.05054 (10)	−0.14886 (4)	0.36963 (6)	0.01449 (17)	
S8	0.63332 (11)	0.29739 (5)	0.79188 (6)	0.02255 (19)	
O1	0.2235 (3)	−0.17383 (12)	0.54846 (15)	0.0180 (5)	
C11	0.7178 (5)	0.3241 (2)	0.6839 (3)	0.0263 (8)	
H13A	0.8305	0.2994	0.6825	0.039*	
H13B	0.7326	0.3792	0.6825	0.039*	
H13C	0.6362	0.3081	0.6280	0.039*	
C10	0.5747 (5)	0.1720 (2)	1.0279 (2)	0.0303 (9)	
H12A	0.5026	0.2156	1.0045	0.045*	
H12B	0.6174	0.1787	1.0960	0.045*	
H12C	0.5051	0.1256	1.0187	0.045*	

### Atomic displacement parameters (Å^2^)

**Table:**

	*U*^11^	*U*^22^	*U*^33^	*U*^12^	*U*^13^	*U*^23^
I2	0.01932 (12)	0.02057 (12)	0.01307 (11)	0.00006 (9)	−0.00063 (8)	0.00219 (8)
I1	0.01529 (11)	0.01492 (11)	0.01614 (11)	−0.00063 (8)	−0.00027 (8)	−0.00382 (8)
C9	0.0150 (16)	0.0153 (17)	0.0153 (16)	−0.0013 (13)	0.0020 (12)	−0.0021 (13)
C1	0.0128 (15)	0.0164 (17)	0.0153 (16)	−0.0002 (13)	0.0014 (12)	−0.0051 (13)
C6	0.0140 (15)	0.0145 (17)	0.0117 (15)	0.0028 (13)	−0.0003 (12)	0.0016 (12)
C4	0.0151 (15)	0.0091 (16)	0.0122 (15)	0.0011 (12)	0.0028 (12)	−0.0019 (12)
C8	0.0142 (16)	0.0163 (17)	0.0156 (17)	−0.0025 (13)	0.0019 (13)	−0.0050 (13)
C2	0.0120 (15)	0.0183 (18)	0.0118 (16)	−0.0014 (13)	−0.0016 (12)	0.0003 (13)
C3	0.0119 (15)	0.0114 (16)	0.0141 (16)	0.0013 (12)	0.0038 (12)	0.0009 (12)
C7	0.0119 (15)	0.0174 (17)	0.0136 (16)	−0.0007 (12)	0.0029 (12)	−0.0011 (13)
C5	0.0103 (15)	0.0164 (17)	0.0165 (16)	0.0033 (12)	0.0037 (12)	0.0009 (13)
S6	0.0201 (4)	0.0142 (4)	0.0148 (4)	−0.0009 (3)	0.0000 (3)	−0.0002 (3)
S4	0.0172 (4)	0.0111 (4)	0.0143 (4)	−0.0004 (3)	−0.0011 (3)	−0.0005 (3)
S2	0.0170 (4)	0.0104 (4)	0.0123 (4)	−0.0004 (3)	−0.0005 (3)	0.0008 (3)
S7	0.0217 (4)	0.0225 (5)	0.0151 (4)	−0.0016 (4)	−0.0021 (3)	−0.0028 (3)
S5	0.0198 (4)	0.0152 (4)	0.0149 (4)	−0.0016 (3)	−0.0003 (3)	−0.0011 (3)
S3	0.0206 (4)	0.0137 (4)	0.0143 (4)	−0.0007 (3)	−0.0023 (3)	0.0011 (3)
S1	0.0175 (4)	0.0101 (4)	0.0150 (4)	0.0002 (3)	−0.0009 (3)	−0.0004 (3)
S8	0.0317 (5)	0.0141 (4)	0.0218 (5)	−0.0030 (4)	0.0036 (4)	−0.0037 (3)
O1	0.0220 (12)	0.0136 (12)	0.0170 (12)	−0.0005 (10)	−0.0016 (9)	0.0008 (9)
C11	0.032 (2)	0.0164 (19)	0.031 (2)	−0.0020 (15)	0.0087 (16)	0.0030 (15)
C10	0.036 (2)	0.036 (2)	0.0183 (19)	0.0172 (18)	0.0010 (15)	−0.0053 (16)

### Geometric parameters (Å, °)

**Table:**

I2—C2	2.080 (3)	C2—S2	1.755 (3)
I1—C1	2.082 (3)	C3—S2	1.736 (3)
C9—C8	1.347 (4)	C3—S1	1.740 (3)
C9—S8	1.751 (3)	C7—S6	1.764 (3)
C9—S6	1.766 (3)	C7—S5	1.763 (3)
C1—C2	1.333 (4)	C5—O1	1.234 (4)
C1—S1	1.749 (3)	C5—S3	1.780 (3)
C6—C7	1.338 (4)	S7—C10	1.810 (4)
C6—S3	1.759 (3)	S8—C11	1.807 (4)
C6—S4	1.765 (3)	C11—H13A	0.9800
C4—C3	1.366 (4)	C11—H13B	0.9800
C4—C5	1.442 (4)	C11—H13C	0.9800
C4—S4	1.756 (3)	C10—H12A	0.9800
C8—S7	1.757 (3)	C10—H12B	0.9800
C8—S5	1.763 (3)	C10—H12C	0.9800
			
C8—C9—S8	125.2 (2)	O1—C5—C4	123.8 (3)
C8—C9—S6	116.8 (2)	O1—C5—S3	122.1 (2)
S8—C9—S6	117.69 (18)	C4—C5—S3	114.0 (2)
C2—C1—S1	117.6 (2)	C7—S6—C9	95.85 (15)
C2—C1—I1	127.7 (2)	C4—S4—C6	95.57 (15)
S1—C1—I1	114.61 (17)	C3—S2—C2	95.67 (15)
C7—C6—S3	124.1 (2)	C8—S7—C10	100.80 (15)
C7—C6—S4	120.0 (2)	C8—S5—C7	95.65 (15)
S3—C6—S4	115.91 (17)	C6—S3—C5	96.70 (15)
C3—C4—C5	120.9 (3)	C3—S1—C1	95.27 (14)
C3—C4—S4	121.3 (2)	C9—S8—C11	101.97 (16)
C5—C4—S4	117.8 (2)	S8—C11—H13A	109.5
C9—C8—S7	126.0 (3)	S8—C11—H13B	109.5
C9—C8—S5	117.6 (2)	H13A—C11—H13B	109.5
S7—C8—S5	116.41 (18)	S8—C11—H13C	109.5
C1—C2—S2	116.5 (2)	H13A—C11—H13C	109.5
C1—C2—I2	128.9 (2)	H13B—C11—H13C	109.5
S2—C2—I2	114.47 (17)	S7—C10—H12A	109.5
C4—C3—S2	120.8 (2)	S7—C10—H12B	109.5
C4—C3—S1	124.4 (2)	H12A—C10—H12B	109.5
S2—C3—S1	114.77 (16)	S7—C10—H12C	109.5
C6—C7—S6	121.5 (2)	H12A—C10—H12C	109.5
C6—C7—S5	124.4 (3)	H12B—C10—H12C	109.5
S6—C7—S5	114.10 (17)		
